# Residents’ Preferences for Household Kitchen Waste Source Separation Services in Beijing: A Choice Experiment Approach

**DOI:** 10.3390/ijerph120100176

**Published:** 2014-12-23

**Authors:** Yalin Yuan, Mitsuyasu Yabe

**Affiliations:** 1 Laboratory of Environmental Economics, Graduate School of Bio-resources and Bio-environmental Science, Kyushu University, Fukuoka 812-8581, Japan; E-Mail: yalin-yuan@agr.kyushu-u.ac.jp; 2 Laboratory of Environmental Economics, Department of Agricultural and Resource Economics, Faculty of Agriculture, Kyushu University, Fukuoka 812-8581, Japan

**Keywords:** waste recycling, waste separation, household waste, multinomial logit model, China

## Abstract

A source separation program for household kitchen waste has been in place in Beijing since 2010. However, the participation rate of residents is far from satisfactory. This study was carried out to identify residents’ preferences based on an improved management strategy for household kitchen waste source separation. We determine the preferences of residents *in an ad hoc sample*, according to their age level, for source separation services and their marginal willingness to accept compensation for the service attributes. We used a multinomial logit model to analyze the data, collected from 394 residents in Haidian and Dongcheng districts of Beijing City through a choice experiment. The results show there are differences of preferences on the services attributes between young, middle, and old age residents. Low compensation is not a major factor to promote young and middle age residents accept the proposed separation services. However, on average, most of them prefer services with frequent, evening, plastic bag attributes and without instructor. This study indicates that there is a potential for local government to improve the current separation services accordingly.

## 1. Introduction

Rapid population growth, urbanization, industrialization, and economic development have resulted in the generation of an enormous volume of solid waste in residential areas throughout the world, particularly in the rapidly growing cities of the developing countries. Separation of waste at the source is admittedly better than the recovery of materials from mixed wastes [1,2,3,4], because it produces cleaner, higher-quality materials as well as reduces the cost of disposal in the long run.

The metropolis of Beijing, in China, covers an area of approximately 1400 km^2^ and has a population of more than 20 million, approximately 86% of whom live in the urban area. The total municipal solid waste (MSW) transported in 2009 was 6.56 million tons, of which organic waste comprised over 60% [5]. However, only 4% of total MSW was composted [6]. In 2010, the local government set targets to compost 30% of the MSW by the end of 2015. Meanwhile, the local government has been undertaking source separation campaigns since 2010.

### 1.1. Program Progress 

Every year hundreds of communities are selected from all over Beijing as pilot communities, and households in these communities are encouraged to separate their kitchen waste from recyclable and other waste. By the end of 2012, this program covered 2412 communities—more than half of all the communities in Beijing. In order to achieve the cooperation of residents and encourage them to conduct source separation, local authorities have made great efforts, such as providing free household kitchen waste containers (half year’s biodegradable plastic bags—one bag per day, and barrels) and hiring separation instructors to guide and help households correctly separate kitchen waste. Besides, they can deliver their waste into community garbage bins just in front of their residential building without time limitation and extra fees.

According to studies, however, the program has made limited progress [7], which is also the finding in a survey conducted in October 2012 by the Beijing-based environmental organization Friends of Nature [8].

### 1.2. Literature Review

Identifying means of successfully conducting source separation is an issue widely studied around the world. Researchers have identified a number of collection systems that help achieve a balance between generators that want their waste collected with minimal inconvenience and disposal operators that need to be given waste in a manner compatible with the treatments they employ. For example, Dahlén *et al*. [9] compared three different collection systems for sorted household waste in Sweden. Gallardo *et al*. [10,11] analyzed collection systems for sorted household waste in cities and towns in Spain. González-Torre and Adenso-Díaz [12] and Gallardo *et al.* [10] concluded that distance between containers and citizens was the key to encouraging more people to properly recycle their household waste. Apart from that, Gomes *et al.* [13] performed economic assessments in Portugal to compare alternative methods of collecting organic waste. Many researchers have also focused on variables that affect selective collection. Bernstad [14] showed that convenient infrastructure for source segregation of kitchen waste is an important factor in household waste recycling in Sweden, and highlighted the need to address this aspect where waste is generated, that is, in the household. Shaw and Maynard [15] conducted a UK-based study that summarized the fundamental design variables for recycling schemes such as the number of separations residents must make at home, provision of collection containers, collection frequency, educational programs, and financial incentives. Notably, to identify household waste-sorting preferences, researchers have widely adopted choice experiments (CEs) in which residents were given an opportunity to choose the waste management system’s components, such as in Japan by Sakata [16], in London by Karousakis [17], and in Poland by Czajkowski [3].

A number of published papers have investigated recycling of source-separated Chinese household waste. For example, Tai *et al.* [18] conducted a comparative analysis on eight major cities in China, which launched MSW source-separated collection in 2000. Zhuang *et al.* [19] proposed a novel source separation method, according to which household waste was classiﬁed as food waste, dry waste, and harmful waste in Hangzhou. Yang *et al.* [20] established a mathematical model of source separation activity, and Deng *et al.* [7] conducted a survey on the status quo of household waste separation in Beijing and conducted simple statistical analysis. Huang *et al.* [21] pointed out the importance of not only financial investment for household kitchen waste sorting and reduction but also of appropriate stakeholders in the context of the wider considerations of creating an overall environment for food waste recycling as a mainstream system. Zhou *et al.* [22] conducted a cost-benefit analysis on a waste-to-market model based on extra bonus for households separating organic wastes in a community with 76 households in Guiyang, southwest China. Yuan and Yabe [23] analyzed Beijing resident’s willingness to pay for the household kitchen waste separation services. However, little attention has been paid to resident attitudes, perceptions, and services preferences, especially the preference heterogeneity of the residents on kitchen waste separation services, which is vital for assisting governments in improving the program and formulating more efficient and sustainable source separation services management policies.

Furthermore, although several studies [3,17,24,25] have recognized that household-level separation is inconvenient for the households since it requires space, time, and effort, exactly how they would like to be compensated for their behavior and inconvenience has not been discussed. 

### 1.3. Purpose and Main Results of This Study

This study would like to estimate an improved household kitchen waste separation services, employing a stated-preference CE method to shed light on residents’ valuation, *via* households’ willingness to accept (WTA) compensation for their source separation behavior of household kitchen waste in the survey area of Beijing City. Moreover, how preference differs between young, middle, and old age groups of respondents and their marginal willingness to accept (MWTA) compensation for the service attributes were also evaluated. 

This study is organized as follows: Section 2 presents the theory underlying the CE method. Section 3 discusses the design and data collection of the CE survey, whose results are reported in Section 4. Section 5 concludes with policy implications.

## 2. Theoretical Background of the Choice Experiment (CE) Method 

The CE approach is an economic and environmental valuation technique. It uses a simulated market to directly elucidate respondent preferences and WTA for presumably improved service options. It has been criticized as, for instance, a means of estimating welfare and deriving value for a sequence of elements implemented by a policy or project, but recently, the application of the CE approach has been extended to various areas owing to its strengths in areas including estimating implicit prices for attributes, welfare impacts for multiple scenarios, and the level of customer demand for alternative “service products” in non-monetary terms, such as tourism [26,27], health care [28,29], wetlands [30], air pollution [31], and many other sites. The CE approach has also been applied in studies related to household waste recycling preferences in many counties [3,16,17,32].

The CE method has its theoretical grounding in Lancaster’s model of consumer choice [33] and its econometric basis in random utility theory [34,35]. Lancaster proposed that consumers derive satisfaction not from goods themselves but from the attributes they provide.

Consider a respondent’s choice of source separation services. The utility for option* i* depends on the attributes (*Z*) of the services and socioeconomic characteristics (*S*) of the respondent and can be expressed as: 

(1) $$U_{in}=V_{in}+\varepsilon_{in}=V\left(Z_{in},S_{n}\;\right)+\varepsilon_{in}$$

The probability that individual* n* will choose option *i* over another option *j* is given as:

(2) $$P_{i}=P(V_{in}+\varepsilon_{in}>V_{jn}+\varepsilon_{jn};\;\forall j\in C)$$

where* C* is the complete choice set. The error terms of the utility function are assumed to be independent and identically distributed (IID) with the property of independence of irrelevant alternatives (IIA). The IIA property states that the probability of choosing an alternative is dependent on the utility of the respective options. The probability of choosing option *i* is estimated with the multinomial logit (MNL) model, which is as follows:

(3) $$P_{i}=\frac{\text{Exp}(V_{in})}{\sum_{j\in C}\text{Exp}(V_{in})}$$

The utility function in linear parameters for the* j* th alternative is specified as follows:

(4) $$V_{jn}=\text{ASC}+\beta_{1}X_{1}+\beta_{2}X_{2}+\ldots +\beta_{k}X_{k}+\gamma_{1}S_{1}+\gamma_{2}S_{2}+\ldots +\gamma_{p}S_{p}$$

where *k* is the number of attributes and *p* is the number of socioeconomic variables. The parameters of β are often not specified and vary with the alternatives in the choice sets, meaning that the impact of a choice-specific variable on the odds of a given option being chosen is the same without the consideration of alternatives. ASC is an alternative-specific constant. It captures any systematic variations in choice observations that are associated with an alternative and that are not explained by the attribute variation or the observed socioeconomic characteristics of the respondents. There are *j* − 1 ASCs in an MNL of *j* options.

## 3. Questionnaire Design

The first step in a CE design is to define the separation services in terms of its attributes and the levels these attributes take. As mentioned in the literature review, the attributes of distances citizens walk, collection frequency, number of separations, educational programs, and incentives are important for promoting citizens’ participation in waste recycling. However, this study only focused on household kitchen waste separation and was based on the current collection system in which bins for kitchen waste are placed in front of individual apartment buildings. This constitutes the shortest distance between containers and citizens. Therefore, collection frequency and time, education, and incentives were the most important service attributes and were chosen as the target attributes of this study. Education is presented as instructor which has been included in the current program. To estimate the influence of incentives, non-cash incentive containers and cash incentive compensation were adopted. Their levels are listed in Table 1. The delivery and collection frequency has two levels, namely once per day and twice per week. The two levels of delivery and collection time are evening and morning. The separation instructor also has two levels of need and no need. The two levels for household kitchen waste container are biodegradable plastic bag and barrel. For the bid range of compensation, considering that few studies have conducted kitchen waste separation behavior valuation surveys, we first designed four levels based on the current household waste cleaning fee of Beijing, which is 3 Chinese Yuan Renminbi (CNY) (US$ 0.48) per household per month, and then tested and adjusted them by the pretested survey. Finally, four levels, that is, 1 CNY (US$ 0.16), 5 CNY (US$ 0.81), 8 CNY (US$ 1.29), and 10 CNY (US$ 1.62) per household per month, are used.

**Table 1:** Attributes of household kitchen waste separation services and their levels used in choice experiment (CM) study.

Attribute	Levels	Description and Coding of Levels
1. Frequency	2	Once per day *vs.* Twice per week
2. Time	2	Morning *vs.* Evening
3. Instructor	2	Need *vs.* No need
4. Container	2	Plastic bag *vs.* Barrel
5. Compensation	4	1 CNY per month per household; 5 CNY per month per household; 8 CNY per month per household; 10 CNY per month per household

CNY = Chinese Yuan Renminbi, 1000 CNY = US$ 161.5.

**Figure 1:**
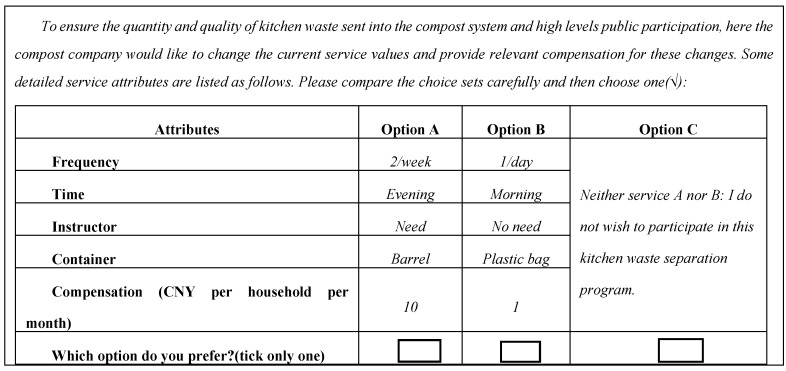
Example of a household kitchen waste separation services choice set.

A number of unique, household kitchen waste recycling service specifications can be constructed from these five attributes; four with two levels and one with four levels (2^4^ × 4 = 64). However, it is difficult to use 64 combinations of profiles in the questions. Therefore experimental design techniques [36] were used with the SPSS conjoint software to obtain an orthogonal design consisting of only main effects, which resulted in 16 pair-wise comparisons of alternative collection services. These were randomly blocked into five different versions, four of which presented three choice sets while one had four. Figure 1 provides a sample choice set. Each set contained two collection service profiles (Option A and B), besides an “opt out” alternative (Option C) where neither A nor B is selected, in which case the respondents were told that separation services would not be provided. Such an opt out option can be considered a status quo or baseline alternative, whose inclusion in the choice set is instrumental to achieving welfare measures that are consistent with demand theory [36,37,38]. Further, respondents were required to choose one out of these three options.

Before the CE question was asked, questions relating to households’ knowledge and awareness of the environment and household kitchen waste management in general, as well as their current situation regarding household kitchen waste generation and separation, were covered. These are very essential to reduce potential information bias because of the limitation of respondents’ knowledge in terms of source separation. At the end of the questionnaire, socioeconomic information on the respondents was collected.

## 4. Data collection 

**Figure 2:**
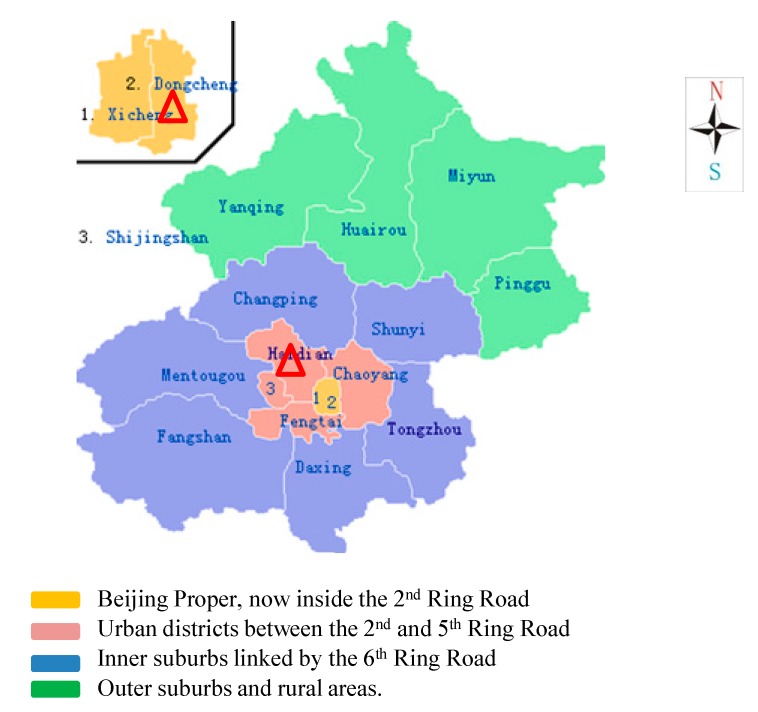
Beijing map and survey area (Source: Google map).

So far, most of the successful cases of economic incentive approaches on household waste recycling are in developed countries such as Netherlands, Belgium, Italy, Luxemburg, Sweden, and especially Denmark and Ireland [39,40]. Thus, we suppose the reason for this is that high- educated and income residents are more prone to support waste sorting than others. Thus, in order to know how behavior change is related to economic persuasion in China, we have to observe several model areas for checking individual recycling activities. As we all know, Beijing is one of the international big cities of China, and has a high percentage of highly-educated and high-income people, especially in the center area. Therefore, this study focused on the center (Beijing proper and urban) area of Beijing City which includes six districts (Figure 2): Dongcheng, Xicheng, Haidian, Chaoyang, Fengtai, and Shijingshan. During this survey, a three-stage sampling technique was employed. The first stage involved the use of purposive sampling techniques whereby two districts—Haidian and Dongcheng—were selected. Because of time and budget constraints, 12 communities (8 from Haidian and 4 from Dongcheng) were randomly selected from all of the 651 pilot communities of Dongcheng and Haidian districts (511 from Haidian and 140 from Dongcheng). During the third stage, respondents were collected *in convenience samples* and interviewed face-to-face in each selected community. It was realized that, each target community has different number of residential buildings with different number of households. In order to get these information and make sure the selected households cover all parts of the area, we visited the community service center of each target community before conducting survey. Besides, to avoid interviewing only a particular group of respondents such as the unemployed, seniors or retirees, the interviewers visited the communities at different times of the day.

The survey took place from 20 June to 1 August 2013. The pretested survey conducted in the first 7 days involved 30 households in three pilot communities not only to test the bid range for the monetary attribute required to estimate the value of the kitchen waste separation services attributes, as mentioned before, but also to ensure that the questionnaire was easy to understand. Finally, five versions of the questionnaire were equally likely to be presented to a given household. At the end of the survey, we approached 401 households and managed to complete 394 interviews for a 98.3% response rate.

## 5. Results 

Table 2 gives descriptive statistics of the main socioeconomic characteristics of the respondents and statistical results from the Beijing Statistic Yearbook [41]. The gender distribution in the samples was about 58% female; higher than the average level. This high percentage may be because the survey was mainly conducted in the daytime, making it more difficult to interview working male respondents. About 57.4% respondents were between 30 to 59 years old. The highest percentage of respondents had attained a four-year university degree or higher level of education (approximate 42%). This is higher than the average Beijing statistical level of ~32%, but is reasonable because central urban areas typically have more highly educated residents than suburban areas. Almost 61% were local people, and the highest percentage of households had three residents. About 70% were living in their own apartment. To estimate respondents’ satisfaction with their household income, we asked them to assess their household’s economic level among urban Beijing households as being low, medium, or high. Less than 33% of the respondents reported their economic level as low. About 12.4% of the respondents had or once had an environmental member in their family, and, according to their self-estimation, no more than 30% always or most times separated their kitchen waste at source. Of the sampled households, 265 were from the Haidian district and 129 from the Dongcheng district. The bias of sample distribution to the statistics in Haidian and Dongcheng districts is probably because the target communities were randomly selected from all pilot communities in the two districts, as mentioned in the section on data collection. The irreconcilable sample distribution is therefore *possibly* understandable. The numbers of respondents for the five versions of the questionnaire were 79, 79, 79, 80, and 77, respectively. 

**Table 2:** Socioeconomic characteristics of the sample and statistics.

Category	Levels	Frequency	%	% ^S^
Gender	Male	166	42.1	50
	Female	228	57.9	50
Age (in years)	≥29	66	16.7	38
	30~59	226	57.4	48
	≤60	102	25.8	14
Local	Yes	240	60.9	63
	Otherwise	154	39.1	37
Household size (in person)	1	26	6.6	22
	2	70	17.8	31
	3	136	34.5	31
	4	76	19.3	9
	≤5	86	21.8	7
House ownership	Own house	277	70.3	--
	Otherwise	117	29.7	
Environmental member	Yes or once	49	12.4	--
	Otherwise	345	87.6	
Economical level	Low	128	32.5	--
	Middle	195	49.5	
	High	71	18	
Education	No high school	125	31.7	44
	High school or equivalent	103	26.1	22
	College or above	166	42.1	32
Separation	Always or most times	115	29.2	--
	Otherwise	279	70.8	
District	Haidian	265	67.3	79
	Dongcheng	129	32.7	21
Version	1	79	20.1	--
	2	79	20.1	
	3	79	20.1	
	4	80	20.3	
	5	77	19.5	
Total	--	394	100	100

^S^ stands for the column of the data calculated by Beijing Statistic Yearbook [41].

Because the Yearbook is limited in that it does not provide exact attribute distribution of the centre urban areas of Beijing. Therefore, the values from the Yearbook results cannot be seen as a precise reference for the survey. However, the Table 2 also shows that the number of people in households in our sample was significantly different to those typically in Beijing, and since this factor is a significant influence on waste produced and recycling rates, we note that although our study might provide some policy suggestions, they would need further investigation.

**Figure 3:**
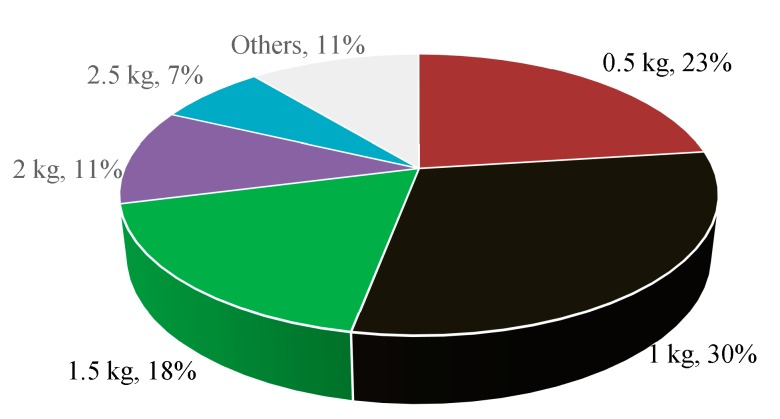
Distribution of the quantity of household kitchen waste on average per day during the last week (*n* = 394).

Besides, we asked the respondents to roughly estimate how many kilograms of kitchen waste each household produced on average per day during the last week (Figure 3). This could help them assess their daily quantity of kitchen waste and the need to take part in its recycling. Approximately 30% of the respondents reported 1 kg, followed by 0.5 kg (about 23%) and 1.5 kg (18%).

### 5.1. Results of Multinomial Logit Model 

Assuming IID error terms with an extreme value type 1 Gumbel distribution, an MNL model can be used to estimate the attribute coefficients. In the choice model analysis, the utility functions depicted in Equation (4) are econometrically estimated using an MNL regression with the software package LIMDEP 8.0 NLOGIT 4.0 [42]. The variables and their coding for the model are presented in Table 3.

**Table 3:** Variables included in multinomial logit model analysis and their levels.

Variables	Definition
*Attribute Variables*	
ASC	Alternative-specific constant (e.g., ASC = 1 if the individual chose Option A or B, and ASC = 0 if the individual chose Option C)
Frequency	1 = Once per day; 0 = Twice per week
Time	1 = Morning; 0 = Evening
Instructor	1 = Need; 0 = No need
Container	1 = Plastic bag; 0 = Barrel
Compensation	1 CNY per month per household; 5 CNY per month per household;
	8 CNY per month per household; 10 CNY per month per household
*Non-attribute Variables*	
Gender	1 = Male; 0 = Female
Education	1 = College or above; 0 = otherwise
Elev1	Economic level: 1 = low; 0 = otherwise
Elev2	Economic level: 1 = middle; 0 = otherwise
Local	1 = local; 0 = otherwise
Separation	Frequency of kitchen waste separation: 1 = always or most times; 0=otherwise

Table 4 shows the regression results with all respondents, and with respondents grouped by age group, that is, young (less than 30 years old), middle (30–59 years old) and old (over 59 years old) age. There are two types of models. First, the basic specification models (M1, M3, M5, and M7) show the importance of the attributes in the respondents’ choice from among the three different options. Models of the second type (M2, M4, M6, and M8) include attribute variables and interaction variables generated from the interaction of seven socioeconomic characteristic with an alternative-specific constant that could improve model fit and remove IIA and IID violations [43]. Besides, the inclusion of these socioeconomic variables also helps to capture the heterogeneity in preferences and estimate the effects of attribute changes on the probability that the improved or base option will be chosen. With the inclusion of the socioeconomic terms, the model fit improves as measured by higher ρ^2^. The Swait-Louviere log-likelihood ratio test rejects the null hypothesis that the regression parameters for these two types of models are equal at the 5% significance level. Thus, the models with socioeconomic variables are better than those with only attributes and will be used for the final interpretations of the CE model. 

**Table 4:** Multinomial logit model estimates for all respondents, and respondents grouped by age group.

Attribute	All Respondents		Age Less than 30		Age 30–59		Age over 59	
	M1	M2	M3	M4	M5	M6	M7	M8
ASC	−0.422 ^***^	−0.831 ^***^	−0.159	0.319	−0.266	−0.987 ^***^	−1.036 ^***^	−1.324 ^***^
Frequency	1.135 ^***^	1.173 ^***^	0.734 ^***^	0.749 ^***^	1.123 ^***^	1.156 ^***^	1.476 ^***^	1.573 ^***^
Time	−0.218 ^**^	−0.223 ^**^	−0.317	−0.296	−0.198 ^*^	−0.201 ^*^	−0.196	−0.192
Instructor	−0.112	−0.109	0.112	0.169	−0.022	−0.048	−0.527 ^***^	−0.482 ^**^
Container	0.224 ^**^	0.229 ^**^	−0.128	−0.194	0.229 ^**^	0.254 ^**^	0.503 ^***^	0.465 ^**^
Compensation	0.008	0.007	0.025	0.026	−0.014	−0.016	0.054 ^*^	0.050 ^*^
ASC·Gender		−0.005		−0.224		−0.074		0.338
ASC·Education		0.483 ^***^		0.639 ^*^		0.470 ^**^		0.287
ASC·Elev 1		0.489 ^**^		−0.098		0.727 ^**^		0.308
ASC·Elev 2		0.273		−0.387		0.406 ^*^		0.222
ASC·Local		−0.709 ^***^		−1.366 ^***^		−0.378 ^**^		−1.047 ^**^
ASC·Separation		1.481 ^***^		0.680		1.379 ^***^		2.240 ^***^
Log likelihood	−1289.46	−1237.31	−220.43	−212.62	−743.19	−716.88	−313.13	−286.12
RsqAdj	0.06	0.09	0.02	0.04	0.06	0.09	0.1	0.17
Iterations completed	5	5	4	5	5	5	5	5
Number of respondents	394	394	66	66	226	226	102	102
Number of choice sets	1259	1259	209	209	727	727	323	323

^*^ Significant at the 10% level; ^**^ significant at the 5% level; ^***^ significant at the 1% level.

Table 4 also presents the coefficients for each of the variables in the eight models. Notice that ASC is negative and significant at the 1% level in M2 with all samples, which means the respondents, on average, prefer the status quo over the proposed separation program. Furthermore, they prefer services with frequent, evening, and plastic bag attributes. Moreover, respondents in different age groups respond differently to the attributes in the choice sets. Young respondents in M4 care only about the collection frequency, middle-age respondents in M6 prefer services with collection frequency, evening collection, and free plastic bag attributes, while elder people as M8 shows prefer services with frequent collection, no instructor, free plastic bag, and compensation attributes. In detail, for the five attributes of the choice sets, frequency has a positive coefficient at the 1% level among the three age groups in which respondents prefer kitchen waste services with greater collection frequency. The time attribute was not a significant incentive for either the young or the old age group, while for the middle age group, it was negatively significant at the 5% level, which means they are prone to deliver kitchen waste in the evening time and prefer evening collection to morning. The insignificant instructor parameter in the young and middle age groups and the negative, significant coefficient in the old age group indicate that the availability of an instructor is not a major incentive for the surveyed residents to accept separation services. The container attribute has little impact on the young respondents’ choice making, while it has a significant and positive effect on middle- and old-age choice behavior. Another important difference between the age groups is that the compensation attribute is not significant in the young and middle age groups, which indicates that low compensation is not a major factor in inducing their preference for the proposed kitchen waste source separation program. On the contrary, it is significant at the 10% level in the last age group.

Concerning the socioeconomic parameters, non-local citizens in the young age group with university or higher degrees are willing to accept source separation services, middle-aged lower-income respondents with university or higher degrees and non-local citizens are more likely to accept these services, and non-local citizens in the old age group prefer kitchen waste collection services. Regarding the separation experience, respondents whose families conduct kitchen waste separation are more likely to support the proposed source separation program.

### 5.2. Marginal Willingness to Accept Estimates 

From Table 4, we can estimate the MWTA compensation of the old age group for significant variables. The MWTA compensation can be estimated from the marginal rate of substitution between the coefficients of the attribute and the monetary variable as follows:

(5) $$MWTA\;Compensation=-\frac{{\text{β}}_{attribute}}{{\text{β}}_{compensation}}$$

Table 5 shows the MWTA compensation results. The estimates are based on the important attributes of the above-60-year-old age group. The results show the MWTA compensation of elder residents for the frequency attribute is about -31.3 CNY (US$ −5.05) per household per month with a 95% confidence interval (CI) between −67.5 CNY (US$ −10.90) and 4.9 CNY (US$ 0.79). This means when the frequency attribute changes from twice per week to once per day, the elder group respondent’s MWTA compensation becomes negative, in other words, their marginal willingness to pay is US$5.05.

**Table 5:** Marginal willingness to accept (MWTA) compensation estimates for age group above 60 in Chinese Yuan Renminbi (CNY).

Attribute	Value	M7	M8
Frequency	Coefficient	−27.4^*^	−31.3^*^
	95% CI	(−56.1, 1.3)	(−67.5, 4.9)
Instructor	Coefficient	9.8	9.6
	95% CI	(−2.6, 22.2)	(−3.9, 23.1)
Container	Coefficient	−9.3^*^	−9.3
	95% CI	(−19.8, 1.2)	(−20.9, 2.4)

^*^ Significant at the 10% level; the 95% confidence interval (CI) was estimated following the procedure suggested by Hanemann and Kanninen [44]; MTWA = marginal willingness to accept.

## 6. Discussion

The results of our analysis suggest a potential for improvement in the source separation services program in Beijing. Unlike Czajkowski *et al.* [3], the results of frequency variable are comparable to Karousakis and Birol [17]. Respondents were more willing to accept services with frequent collection. As regards collection time, this study explores the possibility of evening collection—encouraging households to take out their separated kitchen waste in the evening and deposit it correctly into the community kitchen waste bin, from where it is taken to the disposing site by the collection vehicle. The findings for all age groups showed little or negative interest in the kitchen waste separation instructor. It might thus be worthwhile for the local government to reconsider instructor investment. With regard to the non-cash incentive container factor, the government could consider providing free environment-friendly plastic bags as a long-term incentive. The findings also showed that only old respondents show positive and significant preference for compensation. It would take a great amount of money to install infrastructure (e.g., human resources, garbage bins, transportation facilities, final disposal equipment) in all communities in urban Beijing. Governments could start the program from communities that have a high percentage of older residents and compensate households that separate kitchen waste correctly, which could encourage more separation at the source. By this means, governments can save on costs of achieving separation goals before popularizing the separation campaign in the Beijing area.

## 7. Conclusions

This paper analyzes the preferences of young, middle, and elder age group residents to engage in an improved source separation services program, their socioeconomic determinants, and their MWTA compensation for specific service attributes, namely, delivery frequency, household kitchen waste container, and availability of instructor in Haidian and Dongcheng districts of Beijing City. A CE method was applied to determine the preferences of residents and their MWTA compensation. MNL models were used to analyze resident preferences by age group.

The results show there are differences of preferences on the services attributes between young, middle, and old age respondents. Low compensation is not a major factor to promote young and middle age residents accept the proposed separation services. However, on average, most of them prefer services with frequent, evening, plastic bag attributes and without instructor. The results from this study, however, cannot be generalizable because of the non-typical nature of the sample of residents used. In other words, the results may not be applicable to the entire Beijing area but they may be useful for suggestions to be investigated further for potential policy implications and may serve as a leeway to the next steps in reach in the area regarding WTP for better kitchen waste separation services.

A potential area for further research in the field of household kitchen waste separation could be investigations of residents from more typical households, especially taking household size (number of people in the household) into account. Besides, the low compensation levels set in this study presented some limitations to the MWTA estimations for young and middle-aged respondents considering that this is an exploratory study in the area of waste separation to detect residents’ WTA compensation by the CE method and the unavoidable sampling bias of the pretest survey for compensation attribute levels. Therefore, the compensation levels should be increased in future studies.
